# Partial uniparental isodisomy of chromosome 16 unmasks a deleterious biallelic mutation in *IFT140* that causes Mainzer-Saldino syndrome

**DOI:** 10.1186/s40246-017-0111-9

**Published:** 2017-07-19

**Authors:** Benjamin M. Helm, Jason R. Willer, Azita Sadeghpour, Christelle Golzio, Eric Crouch, Samantha Schrier Vergano, Nicholas Katsanis, Erica E. Davis

**Affiliations:** 1 Division of Medical Genetics and Metabolism, Children’s Hospital of The King’s Daughters, Norfolk, VA 23507 USA; 2 Department of Medical & Molecular Genetics, Riley Hospital for Children at IU Health, Indiana University School of Medicine, Indianapolis, IN 46202 USA; 3 Center for Human Disease Modeling, Duke University Medical Center, Durham, NC 27701 USA; 4 Institute of Genetics and Molecular and Cellular Biology, 67404 Illkirch, France; 5 Department of Ophthalmology, Children’s Hospital of the King’s Daughters, Norfolk, VA 23507 USA; 6 Department of Pediatrics, Eastern Virginia Medical School, Norfolk, VA 23507 USA

**Keywords:** Skeletal ciliopathy, Conorenal dysplasia, Intraflagellar transport, Zebrafish, Whole exome sequencing, Heterodisomy

## Abstract

**Background:**

The ciliopathies represent an umbrella group of >50 clinical entities that share both clinical features and molecular etiology underscored by structural and functional defects of the primary cilium. Despite the advances in gene discovery, this group of entities continues to pose a diagnostic challenge, in part due to significant genetic and phenotypic heterogeneity and variability. We consulted a pediatric case from asymptomatic, non-consanguineous parents who presented as a suspected ciliopathy due to a constellation of retinal, renal, and skeletal findings.

**Results:**

Although clinical panel sequencing of genes implicated in nephrotic syndromes yielded no likely causal mutation, an oligo-SNP microarray identified a ~20-Mb region of homozygosity, with no altered gene dosage, on chromosome 16p13. Intersection of the proband’s phenotypes with known disease genes within the homozygous region yielded a single candidate, *IFT140*, encoding a retrograde intraflagellar transport protein implicated previously in several ciliopathies, including the phenotypically overlapping Mainzer-Saldino syndrome (MZSDS). Sanger sequencing yielded a maternally inherited homozygous c.634G>A; p.Gly212Arg mutation altering the exon 6 splice donor site. Functional studies in cells from the proband showed that the locus produced two transcripts: a majority message containing a mis-splicing event that caused a premature termination codon and a minority message homozygous for the p.Gly212Arg allele. Zebrafish in vivo complementation studies of the latter transcript demonstrated a loss of function effect. Finally, we conducted post-hoc trio-based whole exome sequencing studies to (a) test the possibility of other causal loci in the proband and (b) explain the Mendelian error of segregation for the *IFT140* mutation. We show that the proband harbors a chromosome 16 maternal heterodisomy, with segmental isodisomy at 16p13, likely due to a meiosis I error in the maternal gamete.

**Conclusions:**

Using clinical phenotyping combined with research-based genetic and functional studies, we have characterized a recurrent *IFT140* mutation in the proband; together, these data are consistent with MZSDS. Additionally, we report a rare instance of a uniparental isodisomy unmasking a deleterious mutation to cause a ciliary disorder.

**Electronic supplementary material:**

The online version of this article (doi:10.1186/s40246-017-0111-9) contains supplementary material, which is available to authorized users.

## Background

Genetic medicine in the post-genome era has benefited from increasingly sophisticated methodologies to detect both chromosomal lesions, as well as point mutations across subsets of functionally related genes, or indeed the entire genome [1]. Such approaches are particularly useful for human genetic conditions that are unified by a common cellular basis, but for which extensive phenotypic variability and genetic heterogeneity pose clinical diagnostic challenges. The ciliopathies—multi-systemic disorders caused by dysfunction, altered assembly, and/or altered maintenance of cilia—exemplify a class of inherited disorders which have achieved accelerated molecular discovery in the past ~15 years in part from the advent of whole exome sequencing (WES) and chromosomal array technologies [2–5].

Given the near ubiquitous presence of cilia on different cell types of the vertebrate body plan, it is not surprising that mutations in genes critical for ciliary function can give rise to a broad range of phenotypic features, often characterized by developmental defects in single or multiple systems [6–8]. Some estimates suggest that ~1000 proteins may localize to or be required for ciliary processes [9], thus representing a substantial mutational target. Within the ciliary organelle, macromolecular complexes that perform discrete functions have been characterized biochemically; these include the intraflagellar transport (IFT) complex [10], BBSome [11], and transition zone complex [12], and some phenotype correlations with these complexes have emerged [13]. For example, the skeletal ciliopathies, including Jeune asphyxiating thoracic dystrophy (JATD; MIM 208500), short-rib polydactyly (SRP; MIM 208500), Sensenbrenner syndrome (MIM 218330), and Mainzer-Saldino syndrome (MZSDS; MIM 266920) are enriched for mutations in genes involved in IFT, especially retrograde transport (IFT-complex A). For example, Sensenbrenner syndrome (also known as cranioectodermal dysplasia [14]), a ciliopathy characterized by sagittal craniosynostosis with accompanying facial, skeletal, and ectodermal anomalies, is caused by mutations in the IFT-A genes *IFT43* [15], *WDR35* [16], *IFT122* [17], *WDR19* [18], and *IFT140* [19]. Similarly, the neonatal lethal JATD [20] is hallmarked by a constricted thoracic cage, shortening of the long bones, polydactyly, renal cystic disease, hepatic insufficiency, and retinal degeneration in the individuals who survive past birth. Like Sensenbrenner, JATD can also be caused by mutations in retrograde IFT components, including the motor *DYNC2H1* [21, 22] and IFT-A genes *TTC21B* [23], *WDR19* [18], and *IFT140* [24]. However, neither of these two ciliopathies is caused exclusively by IFT-A dysfunction; for example, JATD can be caused by mutations in the anterograde IFT genes (complex B) *IFT80* [25] or *IFT172* [26]. Furthermore, recessive mutations in a causal gene might not necessarily give rise to a skeletal phenotype, adding further complexity to genotype-phenotype correlations within the skeletal ciliopathies. For example, mutations in *IFT172* can also cause syndromic or non-syndromic retinal degeneration [27].

Here, we report a pediatric case with hallmark retinal, renal, and skeletal phenotypes whose molecular diagnosis was initially unclear. Multi-tiered in vitro and in vivo functional studies suggested that loss of function of *IFT140* is the likely driver, as caused by a combination of mis-splicing and reduction to homozygosity of a missense variant through a uniparental isodisomy mechanism. Together, these results support a diagnosis of MZSDS, represent a rare example of a ciliopathy caused by a non-Mendelian mutational mechanism, and reinforce the need to examine all possible mutation inheritance paradigms when analyzing diagnostic exomes.

## Results

### Case report

**Fig. 1:**
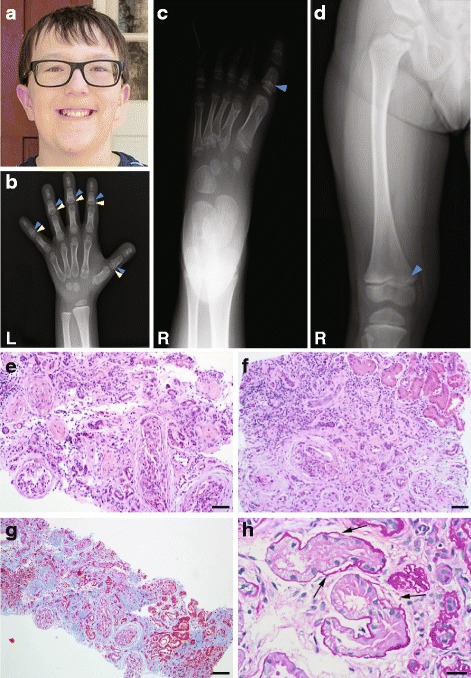
Radiographic and renal features of the proband. **a** Photograph of the index case at 10 years of age. **b** Radiograph of the left (*L*) hand at 5 years and 1 month of age shows cup-shaped metaphyses (*blue arrowheads*), cone-shaped epiphyses (*yellow arrowheads*), and short metacarpals. Bone age is consistent with chronological age. **c** Radiograph of right (R) foot at 5 years and 1 month of age shows shortened proximal phalanges with cup-shaped metaphyses (*blue arrowhead*) and short metatarsals with irregular metaphyses. **d** Radiograph of the right (R) femur showed bilateral coxa valga, broad femoral necks, abnormal femoral metaphyses, and mild bowing of the femoral diaphysis. **e** Renal glomeruli with prominent, periglomerular fibrosis; hematoxylin and eosin (H&E) stain; scale bar, 50 μm. **f** Tubulointerstitial fibrosis, chronic inflammation, tubular atrophy, and focal non-atrophic tubules, (*upper right of panel*); H&E; scale bar 50 μm. **g** Diffuse, severe tubulointerstitial fibrosis and tubular atrophy; Masson’s trichrome stain; scale bar 100 μm. **h** Focal non-atrophic proximal tubules with segmentally thin and duplicated (*arrows*) basement membranes; periodic acid-Schiff stain; scale bar 20 μm

**Fig. 2:**
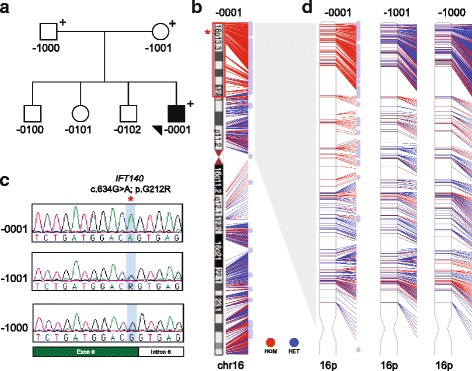
Clinical and research-based genetic analysis. **a** Pedigree of family DM165. **b** Schematic of human chromosome 16. The ~20 Mb region of homozygosity identified in the proband is outlined with a *red rectangle*. The genomic location of *IFT140* is indicated with a *red star*. The 823 variants extracted from the exome data are indicated as homozygous (HOM; *red*) or heterozygous (HET; *blue*); *pale blue circles* indicate homozygous variants of unambiguous maternal origin (*n* = 111). **c** Sequence chromatograms identified a homozygous *IFT140* c.634G>A; p.Gly212Arg mutation that does not segregate in a Mendelian fashion. The mutation location at the exon 6 canonical splice donor site is indicated with a *red star*. **d** Expanded representations of chr16p in each of the proband, maternal, and paternal samples. *Colors* are indicated as described for panel **b**

We consulted a 5-year-old male proband who had a history of complex medical issues, including the following triad of symptoms: retinal dystrophy, acute-onset renal failure, and joint pain (Fig. 1; Additional file 1: Figure S1; Table 1). He was the product of a normal pregnancy with no known teratogenic exposures and was born at term via induced vaginal delivery from his 38-year-old G5 P3-P4 mother. He had a normal birth weight of 3543 g, was measured 48 cm in length, and was the fourth child born to a non-consanguineous couple of northern European ancestry (Fig. 2a). Both parents and all older siblings (two males and one female) were reported healthy. The extended family history was also unremarkable, with the exception of a paternal family history of type 1 diabetes, a paternal grandmother with alpha-1-antitrypsin deficiency, and a maternal cousin who was reported to have hearing loss and retinitis pigmentosa of unknown etiology. The proband reached his developmental milestones on time; although, there were some mild difficulties with visual tracking and speech articulation.

**Table 1:** Phenotype summary

	Case reported here (DM165-0001)	Family V-1 (Perrault et al. 2012) [24]	Family V-2 (Perrault et al. 2012) [24]	Family VII-1 (Perrault et al. 2012) [24]
Sex	M	M	F	M
Age at examination	5 years	4 years; 10 years	2 years; 4 years	Birth; 8 months; 12 months; 18 months
*IFT140* allele 1	c.634G>A (p.Gly212Arg)	c.634G>A (p.Gly212Arg)	c.634G>A (p.Gly212Arg)	c. 2399+1G>T (splice)
*IFT140* allele 2	c.634G>A (p.Gly212Arg)	c.3916dup (p.Ala1306Glyfs*56)	c.3916dup (p.Ala1306Glyfs*56)	c. 634G>A (p.Gly212Arg)
Diagnosis	Mainzer-Saldino syndrome	Mainzer-Saldino syndrome	Mainzer-Saldino syndrome	Jeune syndrome
	(MIM 266920)	(MIM 266920)	(MIM 266920)	(MIM 208500)
Psychomotor development	Dysgraphia	Normal	Normal	Hypotonia
	(HP:0010526),			(HP:0001290),
	Reading difficulties			Poor feeding at birth
	(HP:0010522),			(HP:0008872),
	Adjustment disorder with mixed			Developmental delay
	disturbance of emotions and conduct			(HP:0001263)
	(HP:0100851)			
Cerebral MRI	NA	Normal	Normal	Normal
Retina				
Nystagmus	No	No	No	Yes
				(HP:0000639)
Refraction	High myopia	Myopia	NA	NA
	(HP:0011003)	(HP:0000545)		
Light behavior	Nyctalopia	NA	NA	NA
	(HP:0000662)			
Visual field	NA	Tubular	NA	NA
		(HP:0030588)		
Visual acuity	Mild difficulty with visual tracking	60/200 RE; 120/200 LE with color vision	120/200 RE;100/200 LE	Able to fix and follow
	(HP:0030532)			
Fundus	Retinitis pigmentosa	Retinitis pigmentosa	Retinitis pigmentosa	NA
	(HP:0000510)	(HP:0000510)	(HP:0000510)	
ERG	Rod-cone dystrophy	Severely altered	Severely altered	NA
Craniofacial features	No obvious facial dysmorphisms	Craniosynostosis	Craniosynostosis	Normal
		(HP:0001363),	(HP:0001363),	
		Scaphocephaly	Scaphocephaly	
		(HP:0030799),	(HP:0030799),	
		Facial dysmorphism	Facial dysmorphism	
		(HP:0001999)	(HP:0001999),	
			Microcephaly	
			(HP:0000252)	
Stature	120.2 cm (50th centile) at 6 years and 11 months	Short	Short	Normal
		(HP:0004322)	(HP:0004322)	
Limbs				
Hands	Cone-shaped epiphyses of phalanges	Cone-shaped epiphyses of phalanges	Cone-shaped epiphyses of phalanges	Cone-shaped epiphyses of phalanges
	(HP:0010230),	(HP:0010230),	(HP:0010230)	(HP:0010230)
	Brachydactyly			
	(HP:0011927),			
	Normal bone age			
Feet	Shortened metacarpals	NA	NA	NA
	(HP:0010049),			
	Shortened proximal phalanges			
	(HP:0001831),			
	Shortened metatarsals			
	(HP:0010743),			
	Irregular proximal metatarsal metaphyses			
	(HP:0010630),			
	Brachydactyly			
	(HP:0001831)			
Long bones	Abnormal proximal femoral metaphyses	NA	NA	Trident acetabulum
				(HP:0003170)
	(HP:0003411),			
	Sclerotic changes of the of proximal femoral growth plate			
	(HP:0008797),			
	Broad femoral necks			
	(HP:0006429),			
	Mild bowing of the femoral diaphysis			
	(HP:0002980)			
Other skeletal	Small left joint effusion of the hip	NA	NA	Short thorax
	(HP:0001384),			(HP:0010306),
	Bilateral coxa vara			Short ribs
	(HP:0002812)			(HP:0000773)
Renal features				
Function	Acute-onset renal failure (5 years)	Chronic renal failure (4 years)	Chronic renal failure (9 months)	Elevated urea
	(HP:0001919);	(HP:0000083),	(HP:0000083);	(HP:0000093
		End-stage renal disease (15 years)	End-stage renal disease (3 years)	Proteinuria (18 months)
		(HP:0003774)	(HP:0003774)	(HP:0000093)
Ultrasonography	Pre-transplant renal ultrasound:	Cysts, Small kidneys (-1DS)	1 cyst (cortico-medullar, right	Increased echogenicity
	No evidence of hydronephrosis, Renal cortical medullary parenchymal disease	(HP:0000107)	kidney)	(HP:0004719)
		Hyperechogenicity	(HP:0000107)	
		(HP:0004719),	Hyperechogenicity	
	(HP:0025327)	Cortico-medullary differentiation loss	(HP:0004719),	
			Cortico-medullary differentiation	
	Pre-transplant abdominal ultrasound: Echogenic kidneys bilaterally	(HP:0005565)	loss	
		Growth retardation (-2DS)	(HP:0005565)	
	(HP:0004719)	(HP:0000089)	Growth retardation	
	Post-transplant renal ultrasound: Normal transplanted kidney, normal bladder, atrophic appearance of native kidney		(HP:0000089)	
Biopsy	Diffuse severe tubulointerstitial fibrosis			Non-specific tubulointerstitial nephritis
				(HP:0001970)
	(HP:0005576)			
	Tubular atrophy			
	(HP:0000092			
	Only focal non-atrophic tubules			
Liver	Pre-transplant abdominal ultrasound: Hepatomegaly	Moderate cholestasis	Moderate cholestasis	NA
		(HP:0001396)	(HP:0001396)	
	(HP:0002240)	Hepatic cytolisis with hepatomegaly		
	Possible fatty infiltration			
	(HP:0001397)	(HP:0002240),		
		Portal fibrosis		
		(HP:0006580)		
Other	Pulmonary edema	Bilateral hypoacousia		
	(HP:0100598),	(HP:0000407)		
	Severe hypertension			
	(HP:0000822),			
	Fluid overload			
	(HP:0011105);			
	Mild sclerosis of arterioles			
	(HP:0002634)			

*NA* not available

At 3 years of age, the proband was referred to an ophthalmologist for concerns of nyctalopia, high myopia, and continued poor visual tracking. Fundus exams and electroretinograms (ERG) revealed a rod-cone dystrophy in both eyes with mottling of the retinal pigmentary epithelium and dystrophy of the internal limiting membrane (Additional file 1: Figure S1). The fundus photos show non-specific subretinal hypopigmentation consistent with early onset retinal degeneration typical of rod-cone dystrophies (Additional file 1: Figure S1a). The retinal vasculature appeared attenuated in both eyes. There was no relative afferent pupillary defect and no nystagmus. His high myopia was progressive. At 5 years of age, his refraction was −4.5 and −6.75 diopters in the right and left eyes, respectively. By the age of 8 years old, his best-corrected visual acuity was 20/50 OD and 20/50 OS with glasses and his refraction had increased to −10.25 +2.75 ×106 OD and −11.00 +1.50 ×61 OS. The ERG testing demonstrated reduced cone function under photopic conditions with reduced a-wave implicit time and amplitudes and slowed implicit times for the b-wave (Additional file 1: Figure S1b). Under scotopic testing, both a-waves and b-waves showed slower implicit time and diminished amplitudes (Additional file 1: Figure S1c). The a-wave was substantially delayed, and there were reduced incremental increases in a-wave and b-wave. Audiometric testing was normal bilaterally.

In addition to retinal dystrophy, the proband developed acute-onset renal failure. Initial symptoms included pulmonary edema, severe hypertension, and fluid overload requiring hemodialysis. At 5 years of age, he was transitioned to peritoneal dialysis and remained on dialysis for 5 months, until he received a living related donor kidney transplant. A renal biopsy of the diseased kidney confirmed the cause of renal failure: histological analyses showed diffuse severe tubulointerstitial fibrosis with tubular atrophy and only focal non-atrophic tubules (Fig. 1e, f). Half of the 14 glomeruli examined were obsolescent. The remaining glomeruli appeared partially collapsed and some had segmental capsular adhesion and scarring. Non-obsolescent glomeruli had periglomerular fibrosis with an onion skin-like appearance (Fig. 1g), as evidenced by Masson’s trichrome staining. The tubulointerstitium contained a patchy infiltrate of lymphocytes, and eosinophils were present focally. Periodic acid-Schiff stain (PAS) showed that non-atrophic tubules had segmentally thick and thin tubular basement membranes and segmental duplication (Fig. 1h). Together, these data were suggestive of nephronophthisis [28]; although, we did not detect tubular cysts in the sampled tissue; this may have reflected an absence of the corticomedullary junction in the biopsy. Electron microscopy also showed tubular basement membranes that were focally thick or thin. Arteries were normal and arterioles had mild sclerosis. Except for some segmental entrapment, glomeruli were negative for IgG, IgA, IgM, C3, and C1q by direct immunofluorescence (not shown), ruling out the possibility of C1q steroid-resistant nephrotic syndrome [29].

At the time of the initial evaluation, the proband also reported joint pain with popping of the knees and hips bilaterally. An X-ray bone age study of the hand showed cone-shaped epiphyses of all phalanges with shortened metacarpals and otherwise normal bone age (Fig. 1b). The lower proximal phalanges were short with cup-shaped metaphyses, and the metatarsals were also short (Fig. 1c). Imaging of the knees was normal; however, radiographs of the lower extremities showed bilateral coxa vara, abnormal femoral metaphyses, and sclerotic changes along the metaphyseal side of the femoral growth plate (Fig. 1d). There was broadening of the femoral necks and mild bowing of the femoral diaphysis. Although we could not rule out that some of the joint pain was caused by renal osteodystrophy, the radiographic studies suggested metaphyseal dysplasia. At the time of the most recent examination (6 years and 11 months), the proband weighed 28.8 kg (90th centile), had a height of 120.2 cm (50th centile), and had a fronto-occipital circumference of 52.7 cm (50th centile). A physical exam showed well-healed scars over his abdomen from prior surgeries, with a G-tube in place. His hands showed brachydactyly with shortened distal phalanges, and his facial appearance was not overtly dysmorphic (Fig. 1a). The remainder of the physical examination was otherwise unremarkable.

### Candidate gene analysis identifies *IFT140* as the likely causal gene

Prompted by the acute renal failure of the proband, we conducted clinical panel sequencing of genes implicated in renal conditions (Additional file 1: Table S1). First, we screened six genes that cause nephrotic syndrome or glomerulosclerosis [30–37]. No sequence alterations were identified in *NPHS2*, *LAMB2*, *ACTN4*, or *TRPC6*. In *WT1*, a gene known to be associated with autosomal dominant nephrotic syndrome [32, 36], we identified a heterozygous, intronic variant (c.1099-9T>C); although rare, its frequency in presumably healthy controls in the Exome Aggregation Consortium (ExAC) database indicated that it is likely a benign variant (0.004 minor allele frequency (MAF); 4 homozygotes in 120,852 individuals). Additionally, we detected a rare heterozygous missense change in *NPHS1* (c.1175T>C; p.Leu392Pro) deemed similarly as likely benign due to the presence of healthy homozygous individuals in ExAC (18 of 121,144 samples). Next, we carried out clinical genetic testing of another five genes known to cause the isolated or syndromic renal ciliopathies, nephronophthisis or Senior-Løken syndrome, respectively [38–42]. We identified a missense change that was common in healthy controls (>1% MAF; *SDCCAG8*), three synonymous variants (*GLIS2*, *IQCB1*, *SDCCAG8*), and two deep intronic variants (*IQCB1* and *INVS*), all of which were considered likely benign (Additional file 1: Table S1).

We next considered the possibility that the proband might harbor chromosomal aberrations. We therefore conducted an oligonucleotide (oligo)-single nucleotide polymorphism (SNP) microarray (Affymetrix 6.0). We identified a ~20-Mb region of homozygosity on 16p13.3p12.3 (hg19, chr16:94,807-20,250,946; Fig. 2b). This region encompasses 411 genes, 44 of which are associated currently with disease. When we intersected the clinical synopsis of each disease-related gene with the phenotypes of the proband, we identified a single likely candidate, *IFT140*. This locus encodes a component of the retrograde intraflagellar transport complex [10]. Crucially, mutations in *IFT140* cause a spectrum of ciliopathies, including MZSDS [24], JATD [24], syndromic and non-syndromic retinal dystrophy [43–45], Opitz trigonocephaly C syndrome [46], and Sensenbrenner syndrome [19]. To investigate the possibility that mutations in *IFT140* could contribute to the proband’s clinical presentation, we conducted research-based Sanger sequencing of the 29 coding exons and intron-exon boundaries. We identified a single rare homozygous variant at the exon 6 donor splice site (NM_014714.3: c.634G>A; p.Gly212Arg) in the proband (Fig. 2c). This change is ultra-rare in publicly available control datasets (5/121,320 alleles in ExAC, all heterozygous), and, notably, has been reported in three individuals from two unrelated families with skeletal ciliopathies [24] (Table 1). Segregation of the c.634G>A variant in both parents revealed a Mendelian violation: his mother was a heterozygous carrier, but his father was wild type at the same genomic position (Fig. 2c).

### Functional studies of the IFT140 c.634G>A variant indicate loss of function

**Fig. 3:**
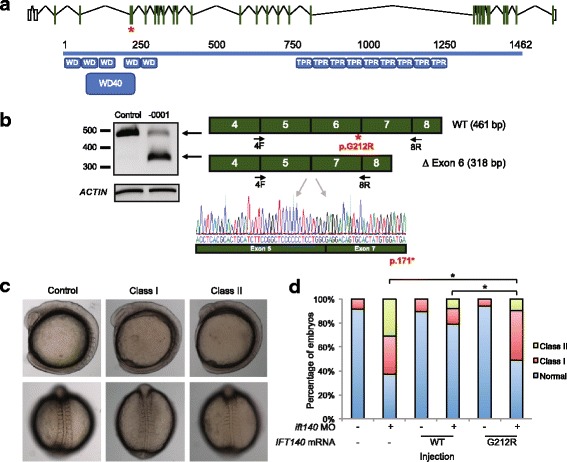
In vitro and in vivo functional analysis of *IFT140* c.634G>A; p.Gly212Arg. **a** Schematic of the human *IFT140* locus at chr16:1,510,427-1,612,110 (hg19; *top*) and translated protein (*bottom*). Exons are depicted as *green boxes*; untranslated regions are shown as *white boxes* (NM_014714.3). Protein schematic (*blue*; NP_055529.2) indicates predicted WD40 repeat (WD40) and tetratricopeptide-like helical domains (TPR). IFT140 c.634G > A; p.Gly212Arg location is indicated with a *red star*. **b** RT-PCR from proband lymphocyte cell lines indicating aberrant mRNA splicing. *IFT140* cDNA was amplified with primers flanking the exon 6 canonical splice donor site (*black arrows*). Sequence chromatograms from purified PCR product indicates that the majority of *IFT140* message is missing exon 6 (*bottom*). **c** Lateral (*top*) and dorsal (*bottom*) views of *ift140* morphants exhibiting gastrulation defects. **d** In vivo complementation studies. Live embryo batches were scored to assay variant pathogenicity. Class I, modest shortening of the body axis and reduction in size of anterior structures; class II, severe shortening of the body axis and decreased anterior structures accompanied by broadening and/or kinking of the notochord and thinning of the somites. To compare pairs of embryo batches, *χ* ^2^ tests were used; *p* < 0.0001 indicated with (*asterisk*)

We considered two mechanisms by which the c.634G>A variant could ablate the function of *IFT140*: (1) disruption of mRNA splicing by abolishing the canonical donor spice site and (2) diminished protein function due to an amino acid alteration of a conserved residue predicted to reside within a WD40 domain (Fig. 3a). We tested both hypotheses.

First, to investigate whether c.634G>A results in aberrant *IFT140* mRNA processing, we established lymphocyte cell lines (LCL) from a whole-blood sample from the proband and we monitored mRNA splicing. We harvested total RNA from proband LCLs and matched control LCLs, reverse transcribed to generate cDNA, and PCR-amplified a 461-bp region spanning exons 4–8 of the canonical *IFT140* transcript. In comparison to the expected sized amplicon in the control sample, we observed two different-sized PCR products in the proband (Fig. 3b). Sequencing revealed that the smaller splice variant was missing the 143-bp *IFT140* exon 6, resulting in a transcript that shifts the reading frame and results in a stop codon at residue 171. The larger mRNA species amplified from proband cDNA was equivalent in size to that of the control, but harbored the c.634G>A mutation (Fig. 3b). Although we do not know whether this correctly spliced mRNA would give rise to a stable or functional protein, the p.Gly212 position is conserved (present in all vertebrates to *Danio rerio*) and p.Gly212Arg is predicted to be probably damaging (PolyPhen-2; score of 1). Together, our in vitro studies revealed the production of two different *IFT140* mRNA species, one resulting in an unambiguous loss of function and another harboring a candidate pathogenic missense variant.

We have shown previously that suppression of both anterograde and retrograde IFT proteins in zebrafish results in gastrulation defects in mid-somite stage embryos [4, 23, 47, 48] and that an assay of missense variants through an in vivo complementation approach [49] is highly concordant with in vitro cilia length complementation studies [23, 47]. We therefore used this approach to test the pathogenic potential of *IFT140* c.634G>A; p.Gly212Arg. Reciprocal BLAST identified a single zebrafish *IFT140* ortholog (59% identity; 76% similarity versus human IFT140), which we suppressed with a previously validated morpholino (MO) [50] that targets the exon 2 splice donor site of the *D. rerio* transcript (GRCz10; ENSDART00000129889.4; Additional file 1: Figure S2a-c). We injected progressively increasing doses of splice-blocking MO (sb-MO) into embryos at the one-to-four-cell stage (3, 6, and 9 ng; *n* = 59–70 embryos/injection batch, repeated twice; Additional file 1: Figure S2d). First, we scored live embryos for gastrulation defects at the nine somite stages according to established scoring criteria (class I is defined as having a shortened anterior-posterior body axis with small anterior structures and mild somite defects; class II is defined as having a severely shortened body axis, severely affected anterior structures, broadening and kinking of the notochord, thinning and widening of the somites, and tail extension defects [4, 23, 47]; Fig. 3c). We observed dose-dependent defects (Additional file 1: Figure S2d). Co-injection of 100 pg WT human *IFT140* mRNA with 8 ng sb-MO resulted in a significant rescue of gastrulation defects (21 versus 62% affected for WT rescue compared to MO alone; *p* < 0.0001; Fig. 3d). Next, we compared the ability of equivalent doses of *IFT140* WT or p.G212R-encoding mRNA to rescue the MO-induced gastrulation defects observed at the mid-somite stage. Embryos injected with the *IFT140* p.G212R*-*encoding change were worse than that of WT rescue, but improved from the MO-injected batches alone, indicating that it is a likely partial loss of function variant (*p* < 0.0001 for all pairwise tests; *n* = 51–78 embryos/injection; repeated twice masked to injection cocktail; Fig. 3d). We saw no significant difference between WT and p.Gly212Arg-encoding mRNA alone. In sum, these data suggest that full-length mRNA encoding IFT140 p.Gly212Arg has impaired function.

### Whole exome sequencing informs the origin of the Mendelian error

The stretch of homozygosity on 16p13 was suggestive of a partial uniparental isodisomy, and identification of a maternal, but not paternal, carrier of the *IFT140* c.634G > A; p.Gly212Arg allele was consistent with this hypothesis. To explore further the basis of this chromosomal event, we conducted trio-based whole sequencing. This experiment offered us the opportunity not only to determine the parental origin of the proband chromosome 16 but also to test whether other variants elsewhere in the exome might contribute to proband phenotype.

We generated 100-bp paired-end reads from exome capture libraries corresponding to each of the proband, mother, and father and conducted variant calling and alignment as described [51]. All three exomes exceeded our quality control thresholds with an average coverage of 117x, and greater than 95% of target bases with ≥20x coverage (Additional file 1: Table S2). We filtered variant data from each individual to retain functional variants (predicted to alter mRNA splicing or protein sequence) with a MAF <1% in two public databases, the National Heart, Lung and Blood Institute (NHLBI) Exome Sequencing project (*n* = 6500 exomes) [52], and the Atherosclerosis Risk in Communities cohort (ARIC; *n* = 2300 exomes) [53]. Next, we compared trio exomes to identify a final candidate gene list harboring rare homozygous, compound heterozygous, X-linked, or de novo variants. Reassuringly, we identified the same homozygous c.634G>A variant in *IFT140* discovered previously by Sanger sequencing. We detected two additional rare variants within the ~20-Mb stretch of homozygosity on 16p13: a missense variant in *ABCA3* (c.4420C>T, p.Arg1474Trp) and another change in *ABCC1* (c.569G>A, p.Cys190Tyr; Additional file 1: Table S3). We also identified compound heterozygous missense variants in *ARHGEF10* (c.1601_1603delTGA, p.Met535del het; c.3967C>T, p.Arg1323Trp het)*.* Although we cannot rule out the possibility that these three genes might contribute to the proband phenotype, they harbor variants of unclear significance that were either not associated previously with any human condition (*ABCC1* and *ARHGEF10*) or have reported roles in human genetic disorders that do not overlap with the proband phenotype (neonatal respiratory distress, *ABCA3* [54, 55]). Thus, *IFT140* remained the most plausible genetic driver of disease.

Finally, we compared chromosome (chr) 16 variant calls across the trio (Fig. 2d). To eliminate sequencing artifacts, we set a stringent single nucleotide variant (SNV) call threshold at ≥10 exome sequencing reads and a quality score >30; 823 of such variants were present in the proband with sufficient coverage to also call the parental genotypes. The majority of variants were uninformative because (1) the genotype was identical across all three individuals, (2) proband genotype was homozygous and both parental genotypes were heterozygous, or (3) proband genotype was heterozygous and one of the two parents was heterozygous. Next, we asked how many SNVs were consistent with the Mendelian violation paradigm observed for *IFT140* c.634G>A. Not only did we identify 76 of such variants within the 16p13 region reported by the oligo-SNP array, but we also observed another 15 variants on the remainder of 16p and 20 variants on 16q that were homozygous in the proband, heterozygous in the maternal sample, and homozygous reference in the father (Fig. 2d). Finally, we tested whether the proband harbored variants that could only have been inherited paternally (not present in the maternal sample); no variants fulfilled these criteria. Importantly, we found an equivalent contribution of rare variation (<1% MAF) from each parent on all other autosomes, excluding the possibility of non-paternity. In sum, our exome data indicate that the proband has a chr16 maternal heterodisomy, with a segmental isodisomy at 16p13 and suggest that an early error in meiosis occurred in the maternal gamete.

## Discussion

Here, we report a case with phenotypic features and a molecular lesion consistent with Mainzer-Saldino syndrome (MZSDS). This disorder, known historically as conorenal dysplasia, is a rare disease characterized by phalangeal cone-shaped epiphyses, chronic renal disease, retinal dystrophy, and mild abnormalities of the proximal femur [56]. Other features may include short stature, hepatic fibrosis, and cerebellar ataxia. MZSDS was first classified as a ciliopathy when recessive mutations in *IFT140* were reported to be causative [24], and it is now known also to be caused by dysfunction of *IFT172* [26]. Three individuals from two unrelated skeletal ciliopathy pedigrees also harbor c.634G>A; p.Gly212Arg, but this allele is in trans with likely null mutations (frameshifting (MZSDS) or canonical splice mutation (JATD)) [24]. Based on this observation, we predict that p.Gly212Arg retains at least partial function. This is supported by reports from IFT gene mutations in humans in which two loss of function mutations are likely incompatible with life past early embryogenesis; recessive null mutations are typically found in *trans* with hypomorphic changes [15–19, 23–27, 43–47, 57, 58]. However, further studies on the protein level will be required to determine whether p.Gly212Arg produces a stable and functional peptide. Although the phalangeal cone-shaped epiphyses, renal phenotypes, and retinal degeneration are consistent between our case and the two reported siblings in family V with MZSDS [3], we note the absence of any overt craniofacial dysmorphisms or craniosynostosis in the case reported here (Table 1). Based on the genetic complexity of the ciliopathies, stochastic, or mutational burden in additional ciliary genes might be contributing to these differences [13].

Our report also represents a rare case of a ciliopathy caused by a segmental uniparental isodisomy. The incidence of uniparental disomy (UPD), in which both homologs of a chromosomal region are inherited from one parent, is not exceedingly rare (estimated ~1:3500 live births [59]) and may not necessarily result in pathology. However, uniparental isodisomy, i.e., the inheritance of a single copy of a parental chromosome or chromosomal segment with concomitant homozygosity, can potentially unmask recessive deleterious alleles inherited from a carrier parent. This phenomenon was first reported in cystic fibrosis in 1988 and is now a recognized, albeit rare, mechanistic basis for inherited disease [60]. There are multiple mechanisms proposed to underlie UPD [61]. Based on the observation of maternal chr16 heterodisomy with isodisomy at 16p13, we consider two possibilities originating from meiosis I error to be the most likely (1) gamete complementation or (2) trisomy rescue. In gamete complementation, fertilization of a disomic chr16 maternal gamete and a nullisomic chr16 paternal gamete would have occurred. In the latter possibility, a disomic chr16 maternal gamete would have been fertilized by a normal male gamete, and a mitotic rescue event would have rebalanced the chromosome number. To determine the likelihood of a trisomy rescue, we evaluated the exome reads covering the 76 homozygous variants on 16p13 in the proband (Fig. 2b, d) for evidence of mosaicism. The majority of variants harbored exclusively alternate reads (49/76; 64%); however, the remainder (27/76; 36%) had 1–3 reference reads at positions annotated as homozygous (Additional file 1: Figure S3). Mosaicism, if any, is likely to be low in the proband, and deeper sequencing would be required to confirm the possibility of a trisomy rescue.

## Conclusions

In summary, we report the successful combination of clinical evaluation and research-based genetic and functional testing to render a molecular diagnosis of Mainzer-Saldino syndrome.

Additionally, we report a rare instance of a uniparental isodisomy unmasking a deleterious mutation to cause a ciliary disorder, highlighting the importance of assessing Mendelian errors in both chromosomal array and trio-based whole exome sequencing studies.

## Methods

### Clinical genetic testing

Panel sequencing was conducted in compliance with the Clinical Laboratory Improvement Amendments (CLIA) regulations as part of standard of care.

Bidirectional Sanger sequencing of coding regions and intron-exon junctions was performed using standard methodology as part of a nephrotic syndrome panel (*NPHS2*, *NPHS1*, *LAMB2*, *WT1*, *ACTN4*, and *TRPC6*; Athena Diagnostics, Worcester, MA) or nephronophthisis panel (*GLIS2*, *IQCB1*, *NPHP3*, *SDCCAG8*, and *INVS*; Prevention Genetics, Marshfield, WI). All test results were reviewed, interpreted, and reported by ACMG-certified Clinical Molecular Geneticists. Chromosome microarray analysis was also performed in a CLIA environment as part of standard of care (Quest Diagnostics, Chantilly, VA). The oligo-SNP array (Affymetrix 6.0) contained over 1.8 million probes, including 900,000 copy-number probes and 900,000 SNP probes with an average inter-probe distance of 700bp; calls were made against the GRCh37/hg19 human genome assembly. Thresholds for genome-wide screening were set at >200 kb for gains and >50 kb for losses, and the threshold used for calling regions of homozygosity was set at >3–5 Mb.

### Bidirectional sequencing of IFT140

Subsequent to informed consent for participation in research, we obtained a blood sample from each member of the trio and extracted DNA from lymphocytes (Gentra Puregene Blood Kit, Qiagen). We PCR-amplified coding regions and intron-exon boundaries and conducted bidirectional cycle sequencing with BigDye v3.1 chemistry on an ABI3730 capillary sequencer. Sequencing reads were aligned to reference and analyzed with Lasergene (DNASTAR) software.

### Lymphocyte cell culture and mRNA splicing studies

B cell lymphocytes were separated from whole-blood samples and were transformed with Epstein-Barr virus (EBV). We cultured cells in RPMI-1640 media supplemented with 10% fetal bovine serum. Wild type cell lines originated from the NHGRI Sample Repository for Human Genetic Research (GM19153; Coriell Institute). We extracted total RNA from LCLs using Trizol reagent (ThermoFisher), and 1 μg was DNAse treated and reverse transcribed using the QuantiTect Reverse Transcription kit (Qiagen). *IFT140* exons 4–8 were amplified and subjected to electrophoresis on a 2% gel, and resulting fragments were gel purified (QIAquick Gel Extraction kit; Qiagen) and sequenced.

### Whole exome sequencing

We generated capture libraries and 100-bp paired-end reads essentially as described [51]. Briefly, we fragmented genomic DNA by sonication, ligated it to Illumina multiplexing PE adapters, and PCR-amplified samples using primers with sequencing indexes. The pre-capture library was then hybridized to biotin-labeled VCRome 2.1 exome probes. Post-capture library DNA was then subjected to sequence analysis on an Illumina Hiseq platform to generate ~9–12 Gb of data. Output data were converted from bcl files to FastQ files by Illumina CASAVA 1.8 software and mapped to the hg19 reference genome by the Burrows Wheeler Alignment (BWA) program. Variant calling was performed using Atlas-SNP and Atlas-Indel [62]. For chr16 variant comparisons, we used Enlis Genome Research software to set thresholds (≥10 exome sequencing reads and a quality score >30) and extract genotype lists for each of the proband, maternal, and paternal exome data. Variant read depth and variant calls were confirmed visually with the Integrated Genomics Viewer (IGV). Data were visualized with PhenoGram (Center for System Genomics, Pennsylvania State University; http://visualization.ritchielab.psu.edu/).

### Zebrafish manipulation and injections

A splice-blocking morpholino (Gene Tools) targeting *ift140* (5′-AGTGATCATGTCTTACCTGCTGCAG-′3) [50] was injected at the indicated concentration into wild-type zebrafish embryos at the one-to-four-cell stage (1 nl/embryo; all injections repeated at least twice, with masked scoring). To validate MO efficiency, we harvested embryos in Trizol reagent at the eight-to-ten-somite stage, extracted total RNA, and conducted RT-PCR studies as described above for human lymphocyte cell lines. To generate human wild-type and mutant mRNA, we first cloned the full-length open reading frame (ORF) (IOH56213; Ultimate ORF, ThermoFisher) into a Gateway-compatible pCS2+ plasmid using LR clonase II (ThermoFisher), linearized with Not I, and performed in vitro transcription with the mMessage mMachine SP6 transcription kit (ThermoFisher). For dose curve or rescue experiments, embryos were scored live at the eight-to-ten-somite stage using previously defined objective scoring criteria [4, 23, 47]. Images were acquired on a Nikon AZ100 microscope facilitated by NIS Elements software. *χ* ^2^ tests were used to perform statistical comparisons of embryo batches in a pairwise manner.

## Additional file

Additional file 1: Table S1. Clinical genetic testing summary. **Table S2.** Whole exome sequencing capture statistics. **Table S3.** Final variant list following trio-based exome analysis (<1% minor allele frequency (MAF), homozygous, compound heterozygous, de novo, or X-linked). **Figure S1.** Fundus imaging and full field electroretinograms indicate retinal dystrophy. a. Fundus imaging of the proband at 5 years 6 months in the right (top) and left (bottom) eyes show mottling of the retinal pigmentary epithelium. b. Photopic (light adapted) testing of cone function demonstrated reduced a-wave implicit time and amplitudes with slowed implicit times for the b-wave. c. Scotopic (dark adapted) testing of rod function demonstrated slower implicit time and diminished amplitudes for both a- and b-waves. The a-wave was substantially delayed with reduced incremental increases in a-wave and b-wave. **Figure S2.** Validation of the *ift140* splice-blocking (sb) morpholino (MO). a. Schematic of the D. rerio *ift140* locus at chr24:37,937,737-38,013,596 (GRCz10; top) and translated protein (bottom). Exons are depicted as green boxes; untranslated regions are shown as white boxes (ENSDART00000129889.4). The sb-MO targets the splice donor of exon (Ex) 2 (red box). Protein schematic (blue) indicates predicted WD40 repeat (WD40) and tetratricopeptide-like helical domains (TPR). b. *ift140* sb-MO induces aberrant splicing of endogenous transcript. *ift140* transcript was evaluated by RT-PCR; embryos were injected with 9 ng MO at the one-to-four-cell stage and harvested for RNA extraction at the mid-somitic stage. Resulting cDNA was amplified with primers flanking the MO target site (arrows in panel a), and migrated on a 2.5% agarose gel. β–actin was used as a control to ensure RNA integrity. c. Chromatograms indicate splicing defects in *ift140* mRNA in morphants. Sequence analysis of purified PCR product indicates that the majority of *ift140* message is missing exon 2 (bottom), which contains the translation initiation codon. Modest amounts of wild type (wt; center) and mRNA containing a 33 nucleotide in-frame intronic insertion (top) are also detectable. d. Representative dose curve of the *ift140* sb-MO. Embryo batches were injected at the one-to-four-cell stage with increasing amounts of *ift140* sb-MO and scored for gastrulation defects at the mid-somitic stage (eight to ten somites). Embryos were scored live according to previously established phenotypic criteria: class I, modest shortening of the body axis and reduction in size of anterior structures; class II, severe shortening of the body axis and decreased anterior structures accompanied by broadening and/or kinking of the notochord and thinning of the somites (See Figure 3; *n* = 59–70 embryos/injection; repeated twice). **Figure S3.** Read depth and variant calls of the homozygous variants called on 16p13. Plot of the 76 maternally inherited homozygous variants identified in the 16p13 region of the proband. Red indicates alternate (alt) reads; blue indicates reference (ref) reads; 49/76 (64%) are comprised only of alternate reads; 27/76 (36%) had one to three reference reads at positions annotated as homozygous. (PDF 20018 kb)

## Abbreviations

bp: Base pair
chr: Chromosome
IFT: Intraflagellar transport
LCL: Lymphocyte cell line
MO: Morpholino
ORF: Open reading frame
SNP: Single nucleotide polymorphism
SNV: Single nucleotide variant
WT: Wild-type
