# Supplementary material for: Partial uniparental isodisomy of chromosome 16 unmasks a deleterious biallelic mutation in IFT140 that causes Mainzer-Saldino syndrome
Source: Hum Genomics. 2017 Jul 19;11:16. doi: 10.1186/s40246-017-0111-9 (PMC5517791; doi:10.1186/s40246-017-0111-9)
Supplement: Additional file 1: Table S1. — Clinical genetic testing summary. Table S2. Whole exome sequencing capture statistics. Table S3. Final variant list following trio-based exome analysis (<1% minor allele frequency (MAF), homozygous, compound heterozygous, de novo, or X-linked). Figure S1. Fundus imaging and full field electroretinograms indicate retinal dystrophy. a. Fundus imaging of the proband at 5 years 6 months in the right (top) and left (bottom) eyes show mottling of the retinal pigmentary epithelium. b. Photopic (light adapted) testing of cone function demonstrated reduced a-wave implicit time and amplitudes with slowed implicit times for the b-wave. c. Scotopic (dark adapted) testing of rod function demonstrated slower implicit time and diminished amplitudes for both a- and b-waves. The a-wave was substantially delayed with reduced incremental increases in a-wave and b-wave. Figure S2. Validation of the ift140 splice-blocking (sb) morpholino (MO). a. Schematic of the D. rerio ift140 locus at chr24:37,937,737-38,013,596 (GRCz10; top) and translated protein (bottom). Exons are depicted as green boxes; untranslated regions are shown as white boxes (ENSDART00000129889.4). The sb-MO targets the splice donor of exon (Ex) 2 (red box). Protein schematic (blue) indicates predicted WD40 repeat (WD40) and tetratricopeptide-like helical domains (TPR). b. ift140 sb-MO induces aberrant splicing of endogenous transcript. ift140 transcript was evaluated by RT-PCR; embryos were injected with 9 ng MO at the one-to-four-cell stage and harvested for RNA extraction at the mid-somitic stage. Resulting cDNA was amplified with primers flanking the MO target site (arrows in panel a), and migrated on a 2.5% agarose gel. β–actin was used as a control to ensure RNA integrity. c. Chromatograms indicate splicing defects in ift140 mRNA in morphants. Sequence analysis of purified PCR product indicates that the majority of ift140 message is missing exon 2 (bottom), which contains the translation initiation codon. Modest am [file 40246_2017_111_MOESM1_ESM.pdf]

Additional File 1
Supplementary Tables

Table S1: Clinical genetic testing summary

Test	Description	Disease Association	Chr. Location (hg19)	Result: nucleotide change	Result: amino acid change	Frequency (ExAC)	Classification
NPHS2 (NM_014625) sequencing	Nephrosis 2, idiopathic, steroid-resistant (podocin)	Nephrotic syndrome/glomerulosclerosis (MIM: 600995)	NA	No mutation detected	NA	NA	NA
LAMB2 (NM_002292) sequencing	Laminin, beta 2 (laminin S)	Nephrotic syndrome (MIM: 614199)	NA	No mutation detected	NA	NA	NA
WT1 (NM_024426) sequencing	Wilms tumor 1	Nephrotic syndrome (MIM: 256370)	11:32417962	c.1099-9T>C	NA	0.004435	Benign
NPHS1 (NM_004646) sequencing	Nephrosis 1, congenital, Finnish type (nephrin)	Nephrotic syndrome (MIM: 256300)	19:36339295	c.1175 T>C	p.Leu392Pro	0.008783	Benign
ACTN4 (NM_004924) sequencing	Actinin, alpha 4	Glomerulosclerosis (MIM: 603278)	NA	No mutation detected	NA	NA	NA
TRPC6 (NM_004621) sequencing	Transient receptor potential cation channel, subfamily C, member 6	Glomerulosclerosis (MIM: 603965)	NA	No mutation detected	NA	NA	NA
GLIS2/NPHP7 (NM_001318918) sequencing	GLIS family zinc finger 2	Nephronophthisis (MIM: 611498)	16:4386814	c.864T>C	p.Tyr288Tyr	0.9796	Benign
IQCB1/NPHP5 (NM_001023570) sequencing	IQ motif containing B1	Senior-Loken syndrome (MIM: 609254)	3:121547527<br><br>3:121526204	c.101-48 T>C<br><br>c.574C>T	NA<br><br>p.Leu192Leu	Not listed<br><br>0.6275	Intronic<br><br>Benign
NPHP3 (NM_153240) sequencing	Nephronophthisis 3 (adolescent)	Nephronophthisis (MIM: 604387)	NA	No mutation detected	NA	NA	NA
SDCCAG8 (NM_006642) sequencing	Serologically defined colon cancer antigen 8	Senior-Loken syndrome (MIM: 613615)	1:243507680<br><br>1:243579112<br><br>1:243493907	c.1473+48dupA<br><br>c.1725G>A<br><br>c.1134A>T	NA<br><br>p.Glu575Glu<br><br>p.Glu378Asp	0.2608<br><br>0.4889<br><br>0.2976	Intronic<br><br>Benign<br><br>Benign
INVS/NPHP2 (NM_014425) sequencing	Inversin	Nephronophthisis (MIM: 602088)	9:103059476	c.3016+48 A>G	NA	0.6803	Intronic
Chromosome microarray (Affymetrix 6.0, 1.8 Mb oligo-SNP)		NA	16p13.3p12.3 (hg19: chr16:94807-20250946) x2 hmz	NA	NA	NA	NA

NA, Not applicable

Table S2. Whole exome sequencing capture statistics

Sample Name	Sample Role	Total Pass Filter (Gb)	% Duplicate Reads	% Total Reads Aligned	Avg Coverage	Reads Hit Target/ Buffer	% Targets Hit	% Base 1+ Coverage	% Base 20+ Coverage	% Base 40+ Coverage
DM165-1000	Father	9	3%	99%	102	77%	99%	99.01%	95.88%	86.75%
DM165-1001	Mother	12	4%	99%	146	77%	99%	99.05%	96.92%	93.28%
DM165-0001	Affected	9	3%	99%	103	77%	99%	99.03%	95.89%	86.66%

Table S3. Final variant list following trio-based exome analysis (<1% minor allele frequency (MAF), homozygous, compound heterozygous, de novo, or X-linked).

Gene	Description	Disease Association	Chr:Location (hg19); rsID	Nucleotide	Amino acid	Zygosity	Inheritance	Total ExAC freq
IFT140	Intraflagellar transport 140 homolog (Chlamydomonas)	Mainzer-Saldino Syndrome (MIM: 266920)	16:1642177 (rs201188361)	c.634G>A exon 6 donor splice site	p.G212R	UPD Hom	Mat	4.121e-05
ABCA3	ATP-binding cassette, sub-family A (ABC1), member 3	Surfactant metabolism dysfunction, pulmonary, 3 (MIM: 610921)	16:2329071 (rs146709251)	c.4420C>T	p.R1474W	UPD Hom	Mat	0.005275*
ABCC1	ATP-binding cassette, sub-family C (CFTR/MRP), member 1		16:16110432	c.569G>A	p.C190Y	UPD Hom	Mat	0
ARHGEF10	Rho guanine nucleotide exchange factor (GEF) 10	Slowed nerve conduction velocity, AD (MIM: 608236)	8:1846642	c.1601_1603 delTGA	p.M535del	Het	AR	0
ARHGEF10	Rho guanine nucleotide exchange factor (GEF) 10	Slowed nerve conduction velocity, AD (MIM: 608236)	8:1905361 (rs201516531)	c.3967C>T	p.R1323W	Het	AR	7.267e-05

***** ≥ 1 Homozygotes present in ExAC (accessed March 2017). Abbreviations: ExAC, Exome Aggregation Consortium; UPD, uniparental isodisomy; Mat, maternal; Pat, paternal; AR, autosomal recessive; MIM, Mendelian inheritance in man.

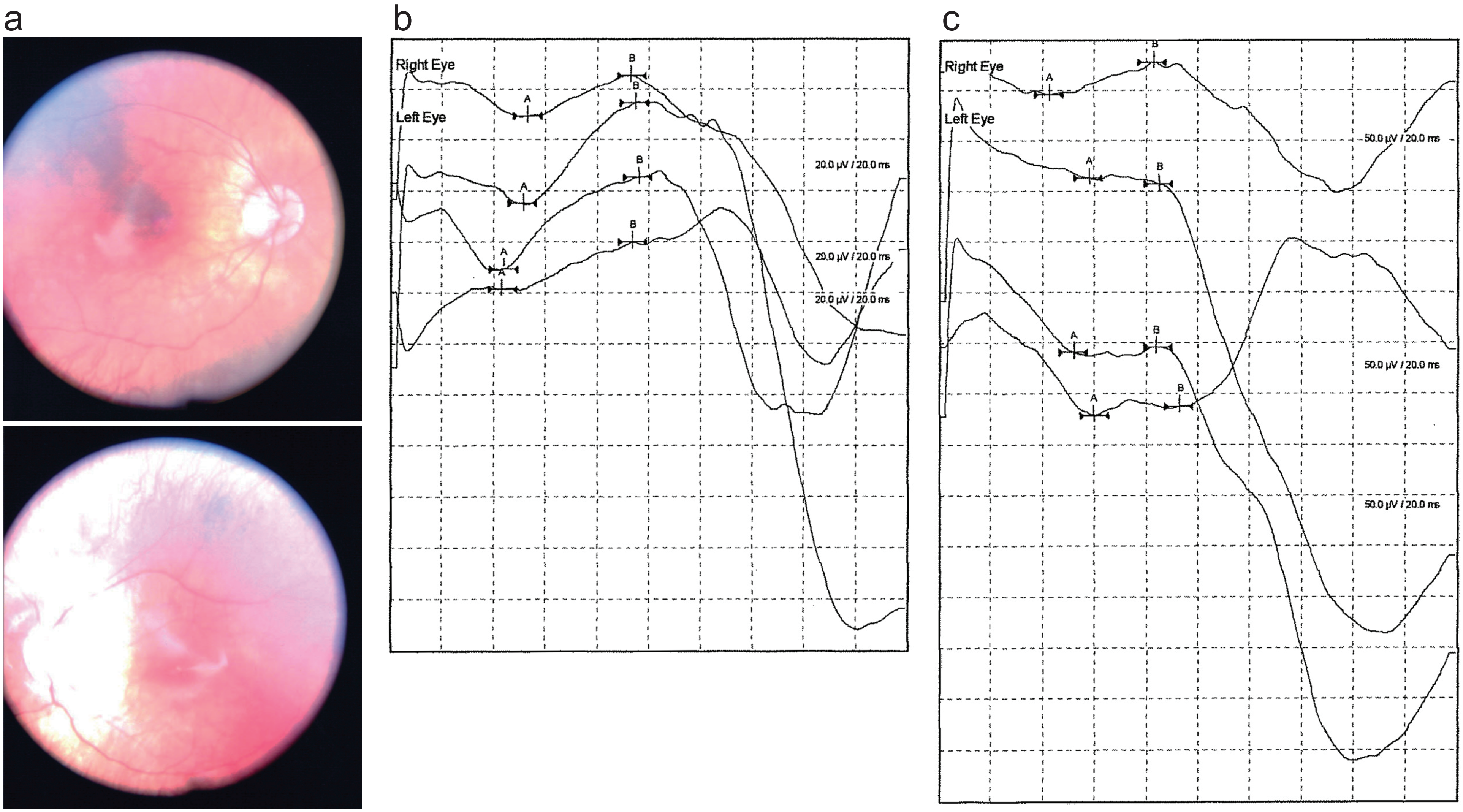

Figure S1

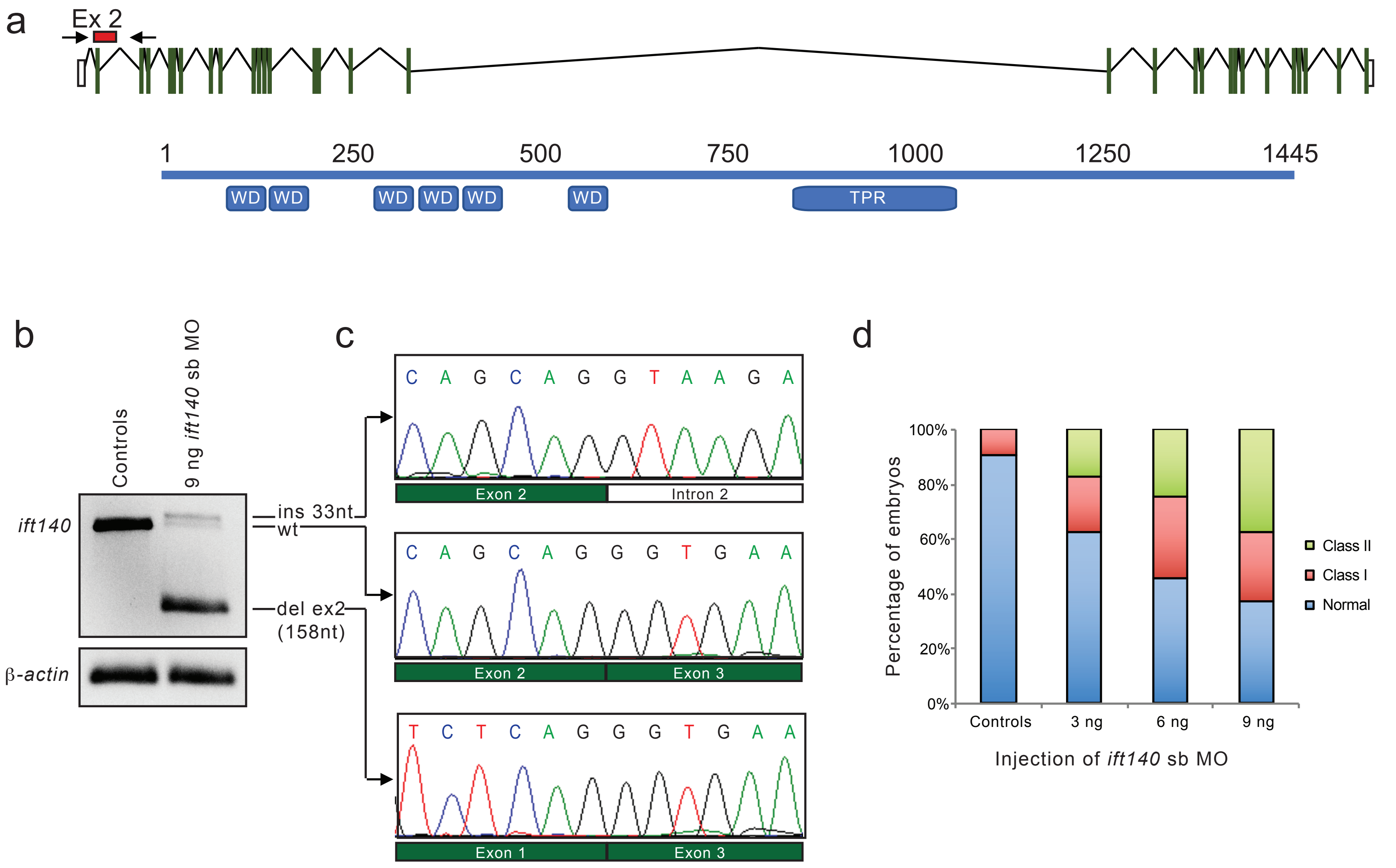
a
Ex 2
1
250
500
750
1000
1250
1445
WD
TPR
b
Controls
9 ng *ift140* sb MO
ift140
β-*actin*
ins 33nt
wt
del ex2
(158nt)
c
C A G C A G G T A A G A
Exon 2
Intron 2
C A G C A G G G T G A A
Exon 2
Exon 3
T C T C A G G G T G A A
Exon 1
Exon 3
d
Percentage of embryos
100%
80%
60%
40%
20%
0%
Controls
3 ng
6 ng
9 ng
Injection of *ift140* sb MO
Class II
Class I
Normal

Figure S2

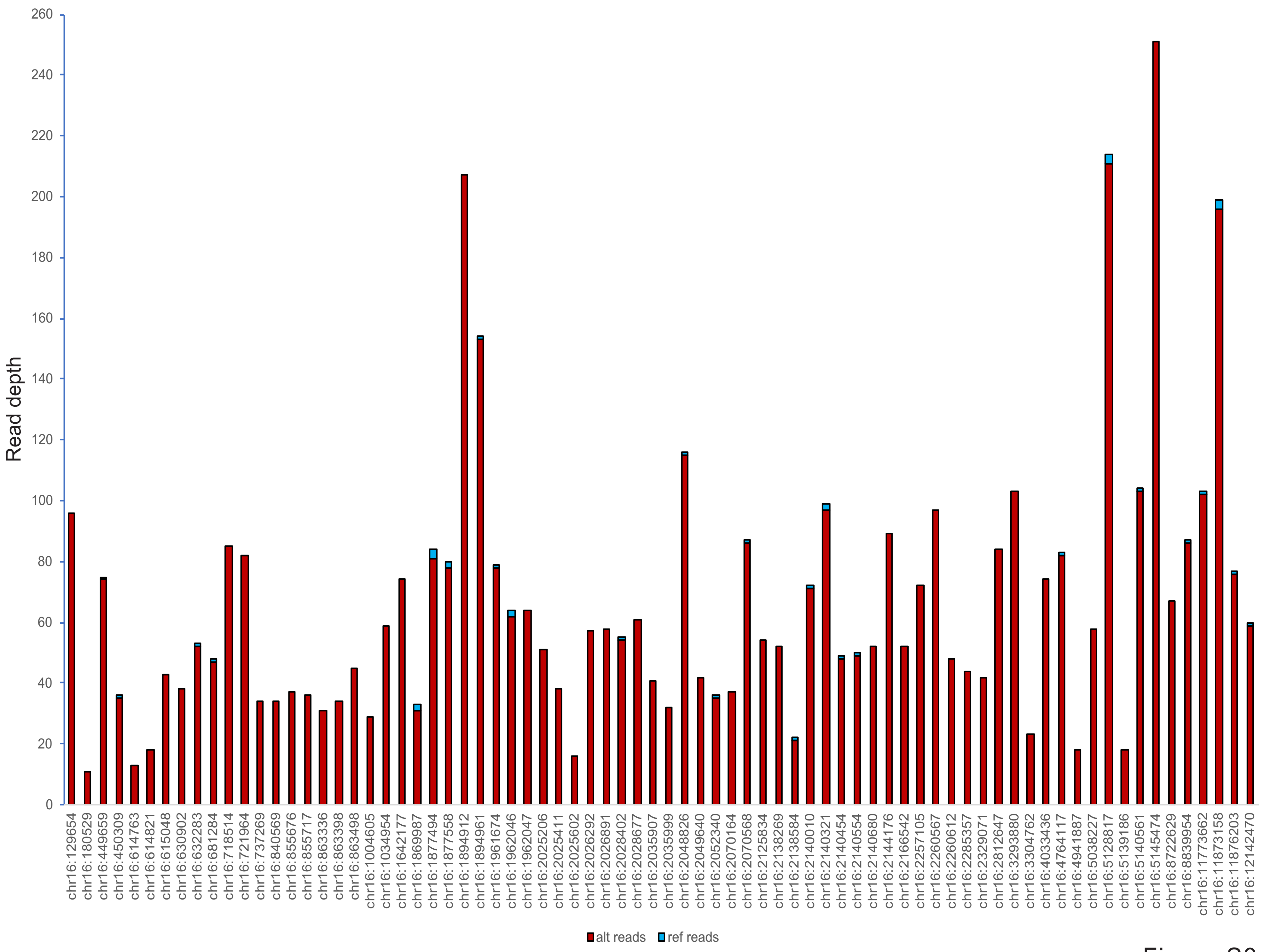
Read depth
260
240
220
200
180
160
140
120
100
80
60
40
20
0
chr16:129654
chr16:180529
chr16:449659
chr16:450309
chr16:614763
chr16:614821
chr16:615048
chr16:630902
chr16:632283
chr16:681284
chr16:718514
chr16:721964
chr16:737269
chr16:840569
chr16:855676
chr16:855717
chr16:863336
chr16:863398
chr16:863498
chr16:1004605
chr16:1034954
chr16:1642177
chr16:1869987
chr16:1877494
chr16:1877558
chr16:1894912
chr16:1894961
chr16:1961674
chr16:1962046
chr16:1962047
chr16:2025206
chr16:2025411
chr16:2025602
chr16:2026292
chr16:2026891
chr16:2028402
chr16:2028677
chr16:2035907
chr16:2035999
chr16:2048826
chr16:2049640
chr16:2052340
chr16:2070164
chr16:2070568
chr16:2125834
chr16:2138269
chr16:2138584
chr16:2140010
chr16:2140321
chr16:2140454
chr16:2140554
chr16:2140680
chr16:2144176
chr16:2166542
chr16:2257105
chr16:2260567
chr16:2260612
chr16:2285357
chr16:2329071
chr16:2812647
chr16:3293880
chr16:3304762
chr16:4033436
chr16:4764117
chr16:4941887
chr16:5038227
chr16:5128817
chr16:5139186
chr16:5140561
chr16:5145474
chr16:8722629
chr16:8839954
chr16:11773662
chr16:11873158
chr16:11876203
chr16:12142470
alt reads
ref reads

Figure S3
